# A Concept Mapping Study of Physicians' Perceptions of Factors Influencing Management and Control of Hypertension in Sub-Saharan Africa

**DOI:** 10.1155/2015/412804

**Published:** 2015-10-13

**Authors:** Juliet Iwelunmor, Sarah Blackstone, Joyce Gyamfi, Collins Airhihenbuwa, Jacob Plange-Rhule, Bamidele Tayo, Richard Adanu, Gbenga Ogedegbe

**Affiliations:** ^1^University of Illinois, Urbana-Champaign, Champaign, IL 61872, USA; ^2^New York University, New York, NY 10016, USA; ^3^Pennsylvania State University, State College, PA 16802, USA; ^4^University of Ghana, Legon, Accra, Ghana; ^5^Loyola University Chicago, Chicago, IL 60660, USA

## Abstract

Hypertension, once a rare problem in Sub-Saharan Africa (SSA), is predicted to be a major cause of death by 2020 with mortality rates as high as 75%. However, comprehensive knowledge of provider-level factors that influence optimal management is limited. The objective of the current study was to discover physicians' perceptions of factors influencing optimal management and control of hypertension in SSA. Twelve physicians attending the Cardiovascular Research Training (CaRT) Institute at the University of Ghana, College of Health Sciences, were invited to complete a concept mapping process that included brainstorming the factors influencing optimal management and control of hypertension in patients, sorting and organizing the factors into similar domains, and rating the importance and feasibility of efforts to address these factors. The highest ranked important and feasible factors include helping patients accept their condition and availability of adequate equipment to enable the provision of needed care. The findings suggest that patient self-efficacy and support, physician-related factors, policy factors, and economic factors are important aspects that must be addressed to achieve optimal hypertension management. Given the work demands identified by physicians, future research should investigate cost-effective strategies of shifting physician responsibilities to well-trained no-physician clinicians in order to improve hypertension management.

## 1. Introduction

Shortages in the health care workforce in Sub-Saharan Africa (SSA) have reached a crisis point [1–3], limiting effective reduction of hypertension related morbidity and mortality rates in patients [4]. In SSA, there are 2.4 million doctors and nurses, which translates to 2 doctors and 11 nursing/midwifery personnel per 10,000 people, compared to 19 doctors and 49 nursing/midwifery personnel per 10,000 in North America [5]. The shortage of health workers is partly due to brain drain or the movement of health personnel in search of a better standard of living, higher salaries, professional advancement opportunities, ready access to advanced technology, and more stable political conditions [2]. These shortages are a multifaceted problem, and improvements are needed if reduction in hypertension related morbidity in SSA is to be achieved.

By the year 2020, available evidence estimates that 75% of all deaths in SSA will be attributable to hypertension [6]. This startling statistic raises important questions about how to best mitigate barriers to optimal hypertension control in the region [7–11]. In SSA countries like Ghana, hypertension is now a major public health concern and the second leading cause of outpatient morbidity in adults aged 45 and above [7, 12–14]. The magnitude of the hypertension problem, including in-depth knowledge of underlying risk factors, remains incompletely understood [4]. Moreover, hypertension is increasing at alarming rates for reasons including urbanization, rapid migration from rural to urban areas, changes in dietary habits, aging of population, and social stress [9, 12, 15, 16]. Poorly managed hypertension also exacerbates the burden of chronic diseases such as stroke, end-stage renal disease, and heart failure, leading to increased morbidity and mortality rates in Africa [17, 18]. Also, knowledge and awareness of hypertension in SSA are alarmingly low, with only 6%–34% of hypertensive adults showing awareness of their status [12, 19, 20]. Although rates of hypertension treatment range from 28% in Ghana [12, 19] to roughly 80% in Nigeria [21, 22], actual rates of patients with maintained blood pressure control are as low as 2% [20].

Most of the existing literature to date in SSA related to hypertension management focuses exclusively on patients [23–25] and sheds little light on physicians' factors or attitudes likely to influence poor patient outcomes. Yet published literature conducted elsewhere suggests that physicians and patients differ substantially in their perceptions, knowledge, and/or attitudes which may lead to conflict or poor patient outcomes [26]. As a result, a better understanding of physicians' perceptions is needed to improve hypertension management in the region and improve quality of care. The purpose of the current study was to determine perceived physician-related barriers and/or facilitators to optimal management and control of hypertension in the region.

## 2. Methods

### 2.1. Participants

A group of physicians attending the Cardiovascular Research Training (CaRT) Institute at the University of Ghana, College of Health Sciences, was invited to participate in this study. The mission of CaRT is to train the next generation of researchers with expertise in cardiovascular epidemiology in SSA, social determinants of cardiovascular disease (CVD), comparative effectiveness research, community-based participatory research, and dissemination and implementation of community- and practice-based interventions targeted at cardiovascular risk reduction. The investigators met with physicians attending CaRT and explained that the goal of the project was to identify factors that influence optimal hypertension control in patients in Sub-Saharan Africa. Following consent, the investigative team asked participants to complete the concept mapping steps below using the Concept Systems Global Max software (Concept Systems, Inc., Ithaca, NY). Inclusion criteria for the study were participants being at least 18 years of age and participating in the CaRT training program. Twelve physicians participated in this study (nine from Nigeria and three from Ghana). Of these, ten were males and two were females. Number of years in practice ranged from six years to sixteen years. Smaller groups are more conducive to concept mapping methods, detailed below, which is the rationale for the small sample size [27].

### 2.2. Concept Mapping Methods

**Figure 1:**
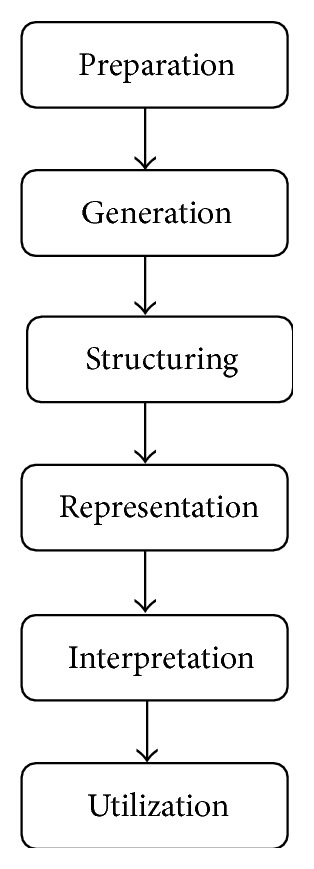
Steps involved in concept mapping procedures.

Concept mapping, a structured, participatory mixed-methods approach to data collection, was used to gather and interpret the ideas generated from physicians in the region. The main steps of concept mapping have been described extensively in the literature [27, 28]. It is a six-step process that integrates group process activities on a topic of interest with several multivariate quantitative analytical tools (multidimensional scaling and hierarchical cluster analysis) to yield both statistical and graphical representation of a conceptual domain [28]. Concept mapping procedures consist of five steps and then utilization of results as follows. (1) Preparation: the researchers outline the research goals and describe that the primary focus is participants' perceptions of factors influencing blood pressure management and control. (2) Generation: the researchers present participants with a focus prompt (e.g., a specific factor that influences blood pressure control is…). Participants generate several items that they believe are relevant to the focus prompt. The investigative team reviews the list to remove duplicates to create a final list of generated ideas. (3) Structuring: participants engage in sorting sessions, in which statements are grouped based on similarities into clusters identified and created by the participants. The sorting provides the data needed to generate clusters of factors that influence hypertension management. (4) Representation: computerized analyses are run and the data are summarized into concept maps. The results of the analysis are shared with participants to be examined and discussed. (5) Interpretation: participants are invited back to interpret the final cluster maps. These interpretation group discussions are designed to address the research goal of exploring factors that influence optimal management of blood pressure control in West Africa. See Figure 1 for an outlined process of concept mapping. Concept mapping is a unique approach that promotes “trustworthiness” in the analysis of qualitative data as it includes participants in all aspects of the research process [28, 29]. Also, the application of the quantitative tools provides structure and reliability to data generated through qualitative techniques [27, 29].

#### 2.2.1. Brainstorming

The following prompt was used to elicit ideas from physicians: “*As a health care provider, a specific factor that affects your ability to control high blood pressure is…*”; brainstorming sessions were conducted online in order to promote candid responses and reduce potential desirability effects. In response to the focus statement, participants were asked to provide concise statements that described factors related to optimal hypertension management and control in patients. A total of 81 statements were generated by participants. The investigative team then distilled these into 54 distinct statements by eliminating duplicate statements, editing statements for clarity, or combining similar statements that reflected physicians' perceptions of factors influencing optimal management of hypertension.

#### 2.2.2. Sorting and Rating

**Figure 2:**
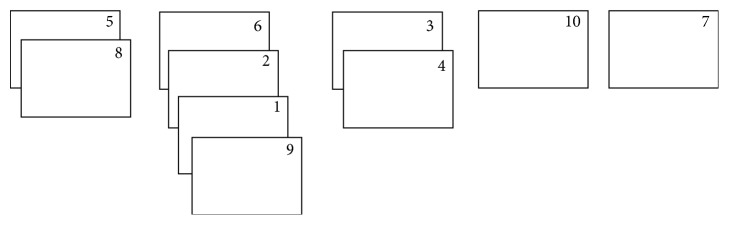
Hypothetical sorting exercise. Participants group statements into different piles based on similarities.

The statements from the brainstorming session were then randomly reordered for concept mapping sorting and rating steps. Physicians worked on an individual basis to sort the statements into conceptually similar groups and were asked to rate the importance and feasibility of statements separately on a 5-point scale. Importance and feasibility were rated on a 5-point scale that ranged from, for importance, 1: relatively unimportant, to 5: extremely important, and, for feasibility, 1: not at all feasible, to 5: extremely feasible, compared to all other statements. For instance, for each statement physicians were asked, “how important is this to hypertension management” and “how feasible is this to address?” See Figure 2 for sample sorting.

### 2.3. Analysis

Concept Systems software was also used for analyzing data generated from the sorting and rating exercise. The software used multidimensional scaling (MDS) to create a two-dimensional “point map” that illustrates the distances between the statements generated for each set of sort data [27]. Aside from being used to structure the sort data, MDS does not independently influence the interpretation of results [29]. The MDS analysis is based on the measurement model that assumes that the relative similarity of objects can be represented in terms of the relative distance between pairs of points [30]. The two-dimensional “point map” is chosen for its ease and interpretability [31]. On the point map, items that are more similar based on participant appear closer together. We also calculated the “stress value” of the point map, which is a measure of how well the MDS solution maps the original data. A lower stress value indicates a better fit of the MDS point map to the original data [29]. Subsequently, we conducted hierarchical cluster analysis of the MDS coordinates and computed average rates of each statement and cluster of statements. This created a cluster map that partitions the statements on the point map into conceptual domains [27]. The maps that result show the individual statements in two-dimensional (*x*, *y*) space with more similar statements located nearer to each other and also show how the clusters partition the space on the map [28, 29]. Since there are no criteria by which a final number of clusters can be selected [29], investigators independently evaluated potential solutions (i.e., 6 clusters and 9 clusters) and agreed on the final model.

### 2.4. Interpretation

In the interpretation phase of concept mapping, we asked physicians to examine different cluster solutions to determine the best number of clusters and grouping of the statements. Clusters were created by participant feedback, based on the distance between items. Items that are closer together illustrate greater degrees of similarity and were sorted together by more people. Each cluster's content was examined for each cluster and discussed amongst participants. The process of determining final clusters was based on participants' subjective preferences. A seven-cluster solution was initially presented to the physicians because the statements within the clusters were conceptually similar to each other. Following further discussion, a four-cluster solution was considered to be visually superior in summarizing the results of the concept mapping. Finally, Go Zone matrices were created based on the final cluster map [27]. Go Zone matrices assign *x*- and *y*-axes to sets of data rated by the physicians where each statement is assigned coordinates based on its respective mean rating. Lines in the Go Zone matrix, which correspond to the mean rating for each axis, divide the graph into four quadrants (low/low, low/high, high/low, and high/high). For instance, items in the top right quadrant are those that were rated as highly important to hypertension management and feasible to address. Following the assumption that high ratings are ideal, the high/high quadrant is considered the Go Zone as it contains those ideas rated most highly on both criteria [32] (i.e., importance and feasibility for addressing patients' blood pressure).

## 3. Results

### 3.1. Cluster Map Solution and Interpretation

**Figure 3:**
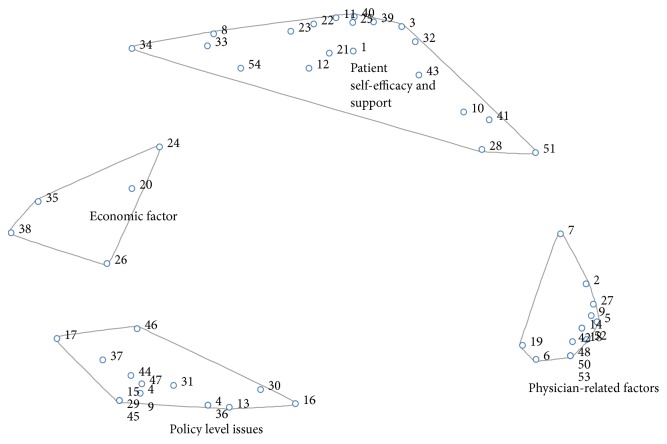
Final cluster map based on participant sorting. Each of the four clusters identified by physicians is represented. Numbers correspond to statements that were sorted into each category. Items that are closer together indicate higher degrees of similarity based on sorting.

The four-cluster concept map generated is presented in Figure 1. Clusters include patient self-efficacy and support factors (20 statements), physician-related factors (14 statements), policy level factors (15 statements), and economic factors (5 statements). See Figure 3. The statements in the clusters are denoted by numbers in the figure and they correspond to the item numbers in Table 1.

Salient statements from the patient self-efficacy clusters included “*lack of patient compliance to therapies: diet, medication, physical activity, and stress management (workplace, home, etc.)*” (item number 3) and “*patients think hypertension is curable after some time on medication*” (item number 43). These statements also reflect physicians' doubt of patients' treatment efficacy. The cluster of physician-related factors reflects barriers and facilitators that physicians themselves may face with helping patients manage and control their hypertension. Two salient statements in the cluster: “*we do not adequately counsel our patients on ways to control their blood pressure*” (item number 9) and “*the failure of physicians to adopt a patient centered model in educating patients about their conditions*” (item number 14), highlight that physicians' attitudes towards hypertension management may be more important than their actual knowledge of the disease. Furthermore, statements in relation to workload (item number 46 “*high patient burden at hypertension clinics*”) may also make it difficult to have good doctor-patient communication or engage in strategies such as patient counseling which would contribute to reduction in high blood pressure rates.

Policy level factors and economic factors reflect physician perceptions of barriers to hypertension management and control operating at the systems level which patients may have little or no control over [33]. For example, statements, such as “*primary healthcare systems are nonfunctional*” (item number 31) and “*the cost and availability of blood pressure drugs*” (item number 38), may affect the hypertension care outcome in patients or contribute to physician inertia that may have anticipated or unanticipated effects with hypertension awareness, treatment, control, and medication adherence in the region [33].

### 3.2. Go Zones

**Figure 4:**
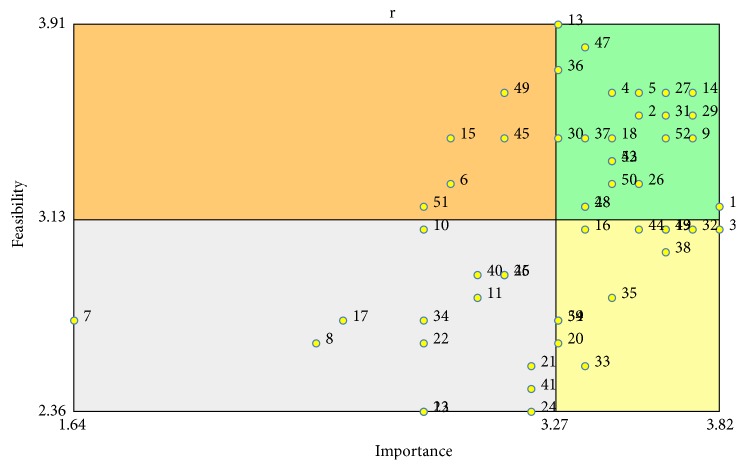
Go Zone analysis on importance and feasibility ratings by physicians. Items in the upper right quadrant represent those ranked highly in terms of importance and feasibility. Items in the lower right quadrant were ranked highly important, but not feasible. Each item number corresponds to the statement number in Table 1.

Figure 4 provides the Go Zones matrices for each of the four clusters. These statements were rated either above or below the mean for both importance and feasibility by the physicians. Of all the statements, the one rated highest for importance was item number 1: “*our patients are in denial and they fail to accept their condition,*” and the highest for feasibility is item number 13: “*we do not have the required equipment to provide the needed care for the management of high blood pressure.*” The statement that was ranked among the lowest for importance is item number 7: “*the age of the patient influences physicians ability to discuss blood pressure management.*” Two items were ranked as not feasible; both were patient related issues and they include item number 22: “*patients experience stress of modern day life,*” and item number 24: “*patients lack resources for follow-up care.*”

## 4. Discussion

Using concept mapping, this study examined physicians' perceptions of factors influencing hypertension management and control in patients in Sub-Saharan Africa. Unlike other methods of qualitative inquiry such as focus groups, concept mapping allows for a shared understanding of factors influencing optimal management and control of hypertension in patients through a wide range of physician generated statements and ideas. In total, 54 statements were generated and grouped into four clusters that reflect patient self-efficacy and support factors, physician-related factors, policy level factors, and economic factors.

Although physicians generally agreed on the importance and feasibility of these statements, it can be surmised from their ratings that two specific factors, namely, patient-related factors (i.e., the patient's ability to come to terms with their diagnosis of hypertension) and economic factors (access to medical resources/equipment for the management of hypertension), were regarded as the most important hurdles that are not too difficult or too costly to surmount. Particularly with regard to patients, failure to accept their condition and denial of hypertension were noted as the most important factor influencing patient adherence to medication and behavioral changes. This may be in part due to the lack of adequate patient counseling on blood pressure management as well as the failure to adopt a patient centered model, which were both noted as barriers to optimal blood pressure management in this study. It might also be inferred that high workload may preclude their engagement of ineffective hypertension management strategies. Indeed, this result complements those of other studies examining chronic disease in Sub-Saharan Africa. Murphy and colleagues [34] found that negative provider interactions affected patients' ability to accept their condition as well as their motivation to engage with providers about behavioral and lifestyle changes needed to manage diabetes. Patients also noted the lack of sufficient counselling and discussed that they wanted providers to spend more time educating them on the disease and management, as they felt ill-equipped to handle it themselves. Additionally, our study found that lack of a functional primary health care mechanism and patients' lack of resources for follow-up care, including regular access to medication, greatly hamper efforts to control blood pressure. This complements the work of Goudge et al. [35], in South Africa, in which the authors found that over half of the households surveyed could not afford regular follow-up care; in some cases families had no income and depended on gifts from friends and other family members to afford health care. Majority of these cases received intermittent treatment at best. Even among households with the means to seek care, only half of the participants were treated regularly for a chronic condition. Weaknesses at the health systems level including poor referral systems, interrupted medication supply, and poor provider-patient interactions were primary barriers to consistent treatment. Interventions addressing these barriers at the provider and health systems level are needed to facilitate optimal maintenance and treatment of hypertension.

Though there is ample evidence that hypertension is an increasing problem in SSA, there is a lack of evidence regarding health care workers' perceptions of factors that influence optimal disease management. Our results complement the few studies that have investigated this topic and are similar to those reported in a recent review of systems level factors influencing hypertension control in SSA [3]. In many parts of SSA, workforce shortage has reached a crisis point and severely limits the ability of physicians to effectively reduce hypertension related morbidity and mortality rates. Workforce shortage and overworked physicians were also noted as barriers in this study. In Parker et al. [32], a survey of 16 health practitioners also identified medical staff shortage as a challenge to the effective management of hypertension at primary care facilities in South Africa. Similarly, an analysis of interviews conducted among patients from 18 community health centers in Cape Town suggested that “*doctors and nurses were working under increasingly difficult circumstances and staff shortages are the main barriers to effectively managing patients with chronic conditions such as hypertension*” 37 g [36]. Similarly, a nurse-led protocol for the management of hypertension in 454 patients over a 26-month period led to a drop of systolic pressure (11.7 mmHg) and diastolic pressure in patients (7.8 mmHg) between baseline and final visits [37]. Task-shifting is one possible solution to the noted barriers by physicians in this study as it would (1) reduce the workload for currently practicing physicians and (2) allow patients to receive more time with health care providers discussing how to properly manage hypertension through appropriate lifestyle changes [38]. As providers in this study noted that patient self-efficacy factors related to acceptance and management of their condition were among the several barriers to optimal hypertension control, more in-depth and longer sessions with health care providers could help patients improve self-efficacy and address concerns related to disease management.

Despite the implications for policy and practice, there are limitations to the current study. The relatively low sample size makes it difficult to generalize findings to other physicians in the region. Furthermore, the concept mapping process itself was very intensive, requiring several hours of participation for brainstorming, sorting of statements, and rating of those statements. The use of the Cardiovascular Research Training Program (CaRT) was extremely helpful as coordinators allocated time during the training which enabled physicians to finish the various concept mapping steps on time. However, the time demand of concept mapping may have prohibited more physicians from participating due to their high workload.

## 5. Conclusion

The findings of our study suggest that hypertension management and control as reflected in the perceptions of physicians is a multifaceted problem. Patient self-efficacy and support, physician-related factors, policy factors, and economic factors are important aspects that must be addressed if reduction in hypertension related morbidity and mortality rate in Sub-Saharan Africa is to be achieved. The strength of this study is the focus on key stakeholders (i.e., physicians) in Sub-Saharan Africa who are often left out of discussions of ways to mitigate the worldwide rising problem of hypertension. Their practical experience allows them insight into a wide range of factors that may impede efforts to reduce hypertension related morbidity and mortality not only at a local but also on a regional and global level. Given the work demands for physicians, future research should explore viable and cost-effective strategies such as task-shifting or the rational distribution of tasks among health care workforce teams in Sub-Saharan Africa.

## Figures and Tables

**Table 1:** Brainstorming statements sorted into their final clusters.

Cluster	Number	Statement (number corresponds to item number on cluster map)
(1) Patient self-efficacy and support	1	Our patients are in denial and they fail to accept their condition
	3	Lack of patient compliance to therapies: diet, medication, physical activity, and stress management (workplace, home, etc.)
	8	The influence of peers or other patients with hypertension
	10	Patients take multiple drugs
	11	Our patients are not conscious of the amount of salt in their diet
	12	The low educational level of our patients
	21	Patients use alternative medication such as herbal, traditional, and spiritual interventions for blood pressure
	22	Poor diet by patients
	23	Patients experience stress of modern day life
	25	Patient adoption of sedentary lifestyle
	28	Our patients do not understand the severity of the disease
	32	Patient nonadherence to medication
	33	Cultural and religious barriers to medication use
	34	Patients lack of family support with hypertension management
	39	The health behaviors of the patients themselves
	40	Patients increasingly patronize fast food
	41	Patients experience side effects of high blood pressure medications
	43	Patients think hypertension is curable after some time on medication
	51	Patients are unable to take the different types of medication given because they are many
	54	The preference for alternative medicines whose providers promise a cure rather than the control and lifetime management offered by physicians

(2) Physician-related issues	2	We do not address medication adherence with our patients
	5	We do not discuss lifestyle activities like physical activity with our patients for blood pressure control
	6	The inability of the physician to empathize and relate to/with his/her clients/patients
	7	The age of the patient influences physicians ability to discuss blood pressure management
	9	We do not adequately counsel our patients on ways to control their blood pressure
	14	The failure of physicians to adopt a patient centered model in educating patients about their conditions
	18	Our communication skills with our patients are poor which makes it difficult for them to comply with our instructions on blood pressure control
	19	Not involving patients in decisions on modalities of treatments
	27	We do not educate patients on the complications of hypertension
	42	Health workers knowledge of blood pressure control is limited
	48	The inability of physicians to identify comorbidities
	50	Physicians lack knowledge and skills which probably will result in lack of confidence to treat these conditions in patients
	52	Not taking into account patients opinion with regard to options for managing their blood pressure
	53	Inertia on the part of clinicians to alter medications to achieve blood pressure control targets

(3) Policy level issues	4	Lack of comprehensive treatment protocols in most centers
	13	We do not have the required equipment to provide the needed care for the management of high blood pressure
	15	The healthcare institutions do not have adequate facilities to provide proper blood pressure services
	16	Lack of adequate hospital follow-up
	17	The long distance between the patient's home and the hospital makes it difficult for follow-up
	29	The use of inaccurate BP monitoring machine
	30	Lack of curricula as the ones available are largely based on infectious disease and little on conditions like hypertension
	31	Primary healthcare systems are nonfunctional
	36	The lack of standard protocol on blood pressure management in our primary health care systems
	37	The availability of the right medication
	44	The nonenforcement of standards of blood pressure treatment
	45	Sometimes, equipment required to measure the blood pressure is either not available or not functional
	46	High patient burden at hypertension clinics
	47	Lack of policy or protocol by the Nigerian health sector on blood pressure control
	49	Lack of standardization of blood pressure measurement in health care settings

(4) Economic factors	20	Patients paying out of pocket
	24	Patients lack resources for follow-up care
	26	Patients use fake drugs
	35	Patients cannot afford the required medication
	38	The cost and availability of blood pressure drugs

Statement numbers correspond to numbered items on cluster map.

## References

[1] Chopra M., Lawn J. E., Sanders D. (2009). Achieving the health Millennium Development Goals for South Africa: challenges and priorities. *The Lancet*.

[2] Kalipeni E., Semu L. L., Mbilizi M. A. (2012). The brain drain of health care professionals from sub-Saharan Africa: a geographic perspective. *Progress in Development Studies*.

[3] Naicker S., Plange-Rhule J., Tutt R. C., Eastwood J. B. (2009). Shortage of healthcare workers in developing countries—Africa. *Ethnicity & Disease*.

[4] Ogedegbe G., Gyamfi J., Plange-Rhule J. (2014). Task shifting interventions for cardiovascular risk reduction in low-income and middle-income countries: a systematic review of randomised controlled trials. *BMJ Open*.

[5] Mayosi B. M. (2013). The 10 ‘Best Buys’ to combat heart disease, diabetes and stroke in Africa. *Heart*.

[6] Kearney P. M., Whelton M., Reynolds K., Muntner P., Whelton P. K., He J. (2005). Global burden of hypertension: analysis of worldwide data. *The Lancet*.

[7] Addo J., Agyemang C., Smeeth L., de-Graft Aikins A., Edusei A. K., Ogedegbe O. (2012). A review of population-based studies on hypertension in Ghana. *Ghana Medical Journal*.

[8] Cooper R. S., Rotimi C. N., Kauftnan J. S., Muna W. F. T., Mensah G. A. (1998). Hypertension treatment and control in sub-Saharan Africa: the epidemiological basis for policy. *British Medical Journal*.

[9] Ibrahim M. M., Damasceno A. (2012). Hypertension in developing countries. *The Lancet*.

[10] Iwelunmor J., Airhihenbuwa C. O., Cooper R. (2014). Prevalence, determinants and systems-thinking approaches to optimal hypertension control in West Africa. *Globalization and Health*.

[11] Unwin N., Setel P., Rashid S. (2001). Noncommunicable diseases in sub-Saharan Africa: where do they feature in the health research agenda?. *Bulletin of the World Health Organization*.

[12] Bosu W. K. (2010). Epidemic of hypertension in Ghana: a systematic review. *BMC Public Health*.

[13] Cook-Huynh M., Ansong D., Steckelberg R. C. (2012). Prevalence of hypertension and diabetes mellitus in adults from a rural community in Ghana. *Ethnicity and Disease*.

[14] Plange-Rhule J., Phillips R., Acheampong J. W., Saggar-Malik A. K., Cappuccio F. P., Eastwood J. B. (1999). Hypertension and renal failure in Kumasi, Ghana. *Journal of Human Hypertension*.

[15] Duboz P., MacIa E., Chapuis-Lucciani N., Boëtsch G., Gueye L. (2012). Migration and hypertension in Dakar, Senegal. *American Journal of Physical Anthropology*.

[16] Hendriks M. E., Wit F. W. N. M., Roos M. T. L. (2012). Hypertension in Sub-Saharan Africa: cross-sectional surveys in four rural and urban communities. *PLoS ONE*.

[17] le Blanc E. S., O'Connor E., Whitlock E. P., Patnode C. D., Kapka T. (2011). Effectiveness of primary care-relevant treatments for obesity in adults: a systematic evidence review for the U.S. Preventive services task force. *Annals of Internal Medicine*.

[18] Lemogoum D., Degaute J.-P., Bovet P. (2005). Stroke prevention, treatment, and rehabilitation in Sub-Saharan Africa. *American Journal of Preventive Medicine*.

[19] Agyemang C., Bruijnzeels M. A., Owusu-Dabo E. (2006). Factors associated with hypertension awareness, treatment, and control in Ghana, West Africa. *Journal of Human Hypertension*.

[20] Houinato D. S., Gbary A. R., Houehanou Y. C. (2012). Prevalence of hypertension and associated risk factors in Benin. *Revue d'Epidemiologie et de Sante Publique*.

[21] Isezuo S. A., Sabir A. A., Ohwovorilole A. E., Fasanmade O. A. (2011). Prevalence, associated factors and relationship between prehypertension and hypertension: a study of two ethnic African populations in Northern Nigeria. *Journal of Human Hypertension*.

[22] Omuemu V. O., Okojie O. H., Omuemu C. E. (2007). Awareness of high blood pressure status, treatment and control in a rural community in Edo State. *Nigerian Journal of Clinical Practice*.

[23] Daniel O. J., Adejumo O. A., Adejumo E. N., Owolabi R. S., Braimoh R. W. (2013). Prevalence of hypertension among urban slum dwellers in Lagos, Nigeria. *Journal of Urban Health*.

[24] Harries T. H., Twumasi-Abosi V., Plange-Rhule J., Cappuccio F. P. (2005). Hypertension management in Kumasi: barriers and prejudice?. *Journal of Human Hypertension*.

[25] Macia E., Duboz P., Gueye L. (2012). Prevalence, awareness, treatment and control of hypertension among adults 50 years and older in Dakar, Senegal. *Cardiovascular Journal of Africa*.

[26] Nam S., Chesla C., Stotts N. A., Kroon L., Janson S. L. (2011). Barriers to diabetes management: patient and provider factors. *Diabetes Research and Clinical Practice*.

[27] Trochim M. K. (2007). *Concept Mapping for Planning and Evaluation*.

[28] Burke S. M., Carron A. V., Eys M. A. (2006). Physical activity context: preferences of university students. *Psychology of Sport and Exercise*.

[29] Rosas S. R., Kane M. (2012). Quality and rigor of the concept mapping methodology: a pooled study analysis. *Evaluation and Program Planning*.

[30] Kruskal J. B. (1964). Multidimensional scaling by optimizing goodness of fit to a nonmetric hypothesis. *Psychometrika*.

[31] Kruskal J. B., Wish M. (2009). *Multidimensional Scaling*.

[32] Parker A., Nagar B., Thomas G., Badri M., Ntusi N. B. A. (2011). Health practitioners' state of knowledge and challenges to effective management of hypertension at primary level. *Cardiovascular Journal of Africa*.

[33] Iwelunmor J., Plange-Rhule J., Airhihenbuwa C. O., Ezepue C., Ogedegbe O. (2015). A narrative synthesis of the health systems factors influencing optimal hypertension control in Sub-Saharan Africa. *PLoS ONE*.

[34] Murphy K., Chuma T., Mathews C., Steyn K., Levitt N. (2015). A qualitative study of the experiences of care and motivation for effective self-management among diabetic and hypertensive patients attending public sector primary health care services in South Africa. *BMC Health Services Research*.

[35] Goudge J., Gilson L., Russell S., Gumede T., Mills A. (2009). Affordability, availability and acceptability barriers to health care for the chronically ill: longitudinal case studies from South Africa. *BMC Health Services Research*.

[36] Labhardt N. D., Balo J.-R., Ndam M., Grimm J.-J., Manga E. (2010). Task shifting to non-physician clinicians for integrated management of hypertension and diabetes in rural Cameroon: a programme assessment at two years. *BMC Health Services Research*.

[37] Kengne A. P., Awah P. K., Fezeu L. L., Sobngwi E., Mbanya J.-C. (2009). Primary health care for hypertension by nurses in rural and urban sub-Saharan Africa. *Journal of Clinical Hypertension*.

[38] Lekoubou A., Awah P., Fezeu L., Sobngwi E., Kengne A. P. (2010). Hypertension, diabetes mellitus and task shifting in their management in sub-Saharan Africa. *International Journal of Environmental Research and Public Health*.

